# COLD-PCR Method for Early Detection of Antiviral Drug-Resistance Mutations in Treatment-Naive Children with Chronic Hepatitis B

**DOI:** 10.3390/diagnostics10070491

**Published:** 2020-07-18

**Authors:** Thuy Thi Bich Phung, Son Van Chu, Son Thien Vu, Hanh Thi Pham, Hang Minh Nguyen, Hoan Dang Nguyen, Ngan Thi Le, Dung Van Nguyen, Phuong Thai Truong, Van Thi Tuong Vu, Anh Thi Van Nguyen

**Affiliations:** 1 Department of Research in Molecular Biology for Infectious Diseases, National Children’s Hospital, Hanoi 10, Vietnam; thuyphung.nhp@gmail.com (T.T.B.P.); minhhang.nhp@gmail.com (H.M.N.); 2 Key Laboratory of Enzyme and Protein Technology, VNU University of Science, Vietnam National University Hanoi, Hanoi 10, Vietnam; sonssc238@gmail.com (S.V.C.); sonthienvu@gmail.com (S.T.V.); hanhpham5514@gmail.com (H.T.P.); 3 Department of Pediatric Gastroenterology-Nutrition-Infectious Diseases, Saint-Paul Hospital, Hanoi 10, Vietnam; hoannd89@gmail.com; 4 Department of Microbiology, Bach Mai Hospital, Hanoi 10, Vietnam; nganmobm@gmail.com (N.T.L.); thaiphuong.vsbm@gmail.com (P.T.T.); tuongvan.bachmai@gmail.com (V.T.T.V.); 5 Center for Tropical Diseases, Bach Mai Hospital, Hanoi 10, Vietnam; dungaids2003@yahoo.com

**Keywords:** COLD-PCR method, hepatitis B virus (HBV), drug-resistance mutation, nucleos(t)ides analogues, treatment-naive children

## Abstract

We investigated Nucleos(t)ide-analogue (NA)-resistance mutations (mt) in 142 treatment-naive children with Chronic Hepatitis B (CHB), using a sensitive co-amplification at lower denaturation temperature (COLD)-PCR with Sanger DNA sequencing. An NA resistance-associated mt in the hepatitis B virus (HBV) reverse transcriptase (RT) was found in 66.2% of the patients, with nonclassical mt contributing the most (64.8%). Significantly higher frequencies of Lamivudine (LMV) and Adefovir dipivoxil (ADF) resistance-associated mt were found in genotypes B and C, respectively (OR_LMV/ADF_: 1495.000; 95% CI: 89.800–24,889.032; *p* < 0.001). Single-point mt associated to LMV and ADF resistance were detected in 59.9% of the tested children with rtV207M (38.0%) and rtN238T (9.9%) being the most frequent. Multiple-point mt were found only in 8 cases (5.6%): 6 children carried double mt (rtV207M + rtL229V; rtV207M + rtI233V; rtV207I + rtV207M × 2 cases; rtV207M + rtS213T; rtN238A + rtS256G) relating to LMV or/and ADF resistance and 3 children carried triple mt (rtL180M + rtM204I + rtN238T; rtV207M + rtS213T + rtS256G) or quadruple mt (rtL180M + rtM204V + rtV207I/M) for LMV-ADF resistance and Entecavir-reduced susceptibility. Our data indicate that significantly higher frequencies of LMV and ADF-associated mutations were found in treatment-naïve children infected with HBV genotypes B and C, respectively. The developed COLD-PCR method and obtained data may contribute to the development of suitable treatments for children with CHB.

## 1. Introduction

Hepatitis B virus (HBV), which could be life threatening by causing liver infection, is a challenging public-health problem worldwide. About 70–90% of pediatric patients with Hepatitis B e-antigen (HBeAg)-positive mothers are perinatally infected and 90–95% of them develop chronic infection [1,2,3,4]. Nucleos(t)ide-analogue (NA) drugs, including Lamivudin (LMV), Adefovir dipivoxil (ADF), Entecavir (ETV), Telbivudine (LdT), and Tenofovir disoproxil fumarate (TDF), have been recommended by international guidelines for suppressing HBV replication in patients with Chronic Hepatitis B (CHB). In general, antiviral therapy is recommended to HBeAg-positive patients with HBV DNA ≥ 20.000 IU/mL (equivalent to 1.10^5^ copies/mL) and HBeAg-negative patients with HBV DNA ≥ 2000 IU/mL (equivalent to 1.10^4^ copies/mL) [5,6,7,8,9]. NA drugs target the viral reverse transcriptase (RT), which catalyzes the reverse transcription of the pre-genomic RNA intermediate, thereby forming the DNA genome [10].

However, the use of NA for prolonged time induces drug-resistance mutations (mt) in the HBV population, including in their RT [11,12]. In terms of resistance development, the NA can be classified as drugs with low (LMV, ADF, and LdT) and high genetic barriers (ETV and TDF) [13,14]. Besides the mt emerging during treatment, the presence of preexisting drug-resistance mt further reduces the efficacy of the treatment. A number of studies have shown that natural HBV RT mt exists even in treatment-naive adult patients with CHB [15,16,17,18]; however, to the best of our knowledge, detection of NA-drug-resistance mt in treatment-naive HBV-infected children has not yet been or is rarely reported. This lack of information raises a question about cost and effectiveness of treatment in pediatric patients if doctors just follow international or regional guidelines without testing for HBV NA-drug resistance mt. According to the American Association for the Study of Liver Diseases’ (AASLD) 2018 Hepatitis B Guidance [9], ETV is ranked as the preferred drug whereas LMV is less preferred for the treatment of CHB in children between 2 and 12 years of age. Likewise, for the treatment of CHB in children above 12 years of age, treatment using TDF is preferred to that of ADF. Thus, sensitive detection of drug-resistance mt is beneficial for treatment efficacy and helps reduce the economic burden by preventing the use of unnecessary medicines. However, drug-resistance mt might exist in a minority within a hetero-viral population, making their early detection difficult. Thus, there is an urgent need for such a sensitive method, which would improve the monitoring of patients and would prompt the decisions to modify their therapeutic regimes. PCR amplification of HBV DNA followed by direct sequencing of the purified amplicons is the gold standard for detecting drug-resistance mt. Because conventional PCR (combined Sanger sequencing) cannot detect minor variants (<20%) [19], Li et al. [20] have developed a highly sensitive method, co-amplification at lower denaturation temperature-PCR (COLD-PCR), which relies on manipulation of the denaturation. The principle of COLD-PCR is based on a critical denaturation temperature (T_c_) for each DNA sequence that is lower than its melting temperature (T_m_). PCR amplification efficiency for a DNA sequence drops dramatically if the denaturation temperature is set below its T_c_. The developed COLD-PCR does not need additional reagents or equipped light cyclers and, thus, can easily replace conventional PCR and, at the same time, improve the mutation detection sensitivity limit of downstream Sanger DNA sequencing, enriching low-level variants (<5%) within a mixture of wild-type (wt) and mt sequences. The reliability of COLD-PCR has been validated by ultradeep pyrosequencing, and COLD-PCR has been successfully applied for detection of several important NA-resistance mt in HBV [21,22]. The aim of this study was to develop a modified COLD-PCR method that identifies most of the known HBV NA-resistance mt and to apply it on clinical samples to describe the current frequencies of these mt in treatment-naive HBV-infected children. These data before the start of treatment may contribute to clinical decision making suitable for children patients having CHB.

## 2. Materials and Methods

### 2.1. Collection of Patients Samples

The study included 142 treatment-naive chronically infected children with HBV (serum levels ≥ 6.10^5^ IU/mL, mainly in the range of 1.10^7^–1.10^9^ IU/mL) from the National Children’s Hospital, Saint-Paul Hospital, and Bach Mai Hospital, Hanoi, Vietnam. An approval from the medical ethics council No. 39/BVNTW-VNCSKTE was received on 9 January 2019 at the Vietnam National Children’s Hospital, and the study was conducted between January 2019 and July 2019. Collection of patients-blood samples was performed in accordance with research ethics regulations in medicine. Parents or legal guardians of patients signed consent forms after they were provided with sufficient information on the research and confidentiality of personal information, allowing patient participation in the study.

### 2.2. Design of Primers for Amplification of Specific Sequences Containing NA Resistance mt

An HBV genome homology database was created based on 32 reference sequences belonging to 8 genotypes (A–H) and was retrieved from National Center for Biotechnology Information (NCBI). Primers for PCR amplification of nucleotides (nt) 532–929, corresponding to the B, C, D, and E domains and containing major NA-drug resistance mt in HBV RT such as rtV173L, rtL180M, rtA181V/T, rtT184G/L, rtA194T, rtS202I/G, rtM204I/V, rtN236T, and rtM250I/V [23], were designed as follows:

Forward-HBV: 5′-TCCTGCTCAAGGAACCTCTATG-3′ (nt 532–553)
Reverse-HBV: 5′-TGTACAATATGTTCCTGTGG-3′ (nt 910–929).

For COLD-PCR amplification of the P1-fragment (nt 594–788), containing the B and C domains, the following primers were used:

Forward-HBV-P1: 5′-CACCTGTATTCCCATCCCATC-3′ (nt 595–615)
Reverse-HBV-P1: 5′-AGGGACTCAAGATGTTGTACA-3′ (nt 768–788).

For amplification of the P2-fragment (nt 732–929), containing D and E domains, the following primers were designed:

Forward-HBV-P2: 5′-CAGTTATATGGATGATGTGGTATTGG-3′ (nt 732–757)
Reverse-HBV-P2: 5′-TGTACAATATGTTCCTGTGG-3′ (nt 910–929).

The size of P1 and P2 fragments were intentionally designed to be <200 nt, as recommended previously [21,22], to ensure the difference in melting temperature between homoduplex (mt-mt; wt-wt) and heteroduplex (mt-wt).

### 2.3. Conventional PCR Combined with Sanger DNA Sequencing

Extraction of HBV DNA was performed using the QIAamp DNA Blood Mini Kit (Qiagen, Hilden, Germany) with 200 µL of serum. For the amplification of the individual DNA sequences, 5 µL of the DNA samples was added to a 20 µL master mix of 2X TOPsimple™ PreMIX-HOT (Enzynomics, Incheon, Korea) containing the respective primers. The PCR conditions were set as follows: 95 °C for 10 min; 45 cycles of 95 °C for 15 s, 60 °C for 20 s, and 72 °C for 20 s. PCR was performed on GeneAmp^®^ PCR System 9700 (Applied Biosystems, Foster, CA, USA), and then the amplified 397 bp-products were checked by agarose-gel electrophoresis before sending for Sanger DNA sequencing (1st Base Axil Scientific Pte, Singapore).

### 2.4. Cloning and Synthesis of RT-HBV wt and Representative mt Controls

Ligation of the amplified wt RT-HBV and the pTOP TA V2 vector was performed using TOP cloner™ TA kit (Enzynomics, Incheon, Korea), and the recombinant plasmid was used as wt control. The mt controls contained representative mt locating randomly at P1 and P2 fragments: rtA194T (G709A), rtM204I (G741T), and rtN236T (A836C) in pUC19 plasmid (PhuSa Biochem, Can Tho, Vietnam). 

### 2.5. Optimizing COLD-PCR Conditions and Evaluating Sensitivity of COLD-PCR Assay Combined with Sanger DNA Sequencing

**Figure 1:**
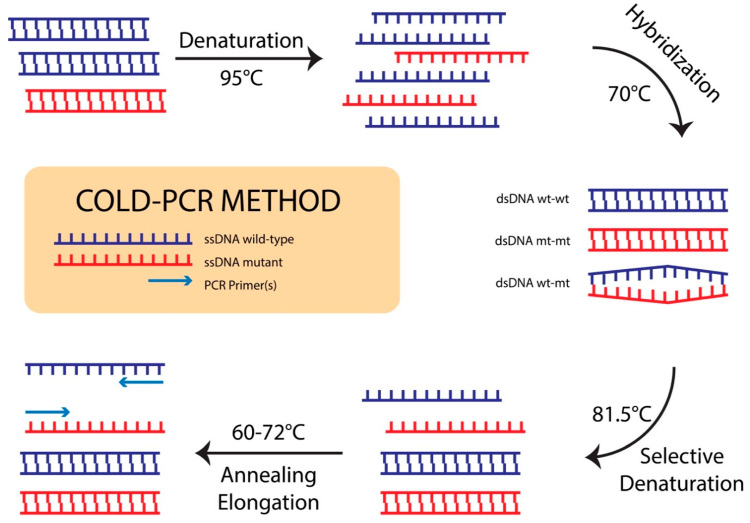
Schematic workflow for co-amplification at lower denaturation temperature (COLD)-PCR: Several rounds of conventional PCR cycles produce the initial material for the target amplicons. After denaturation at 95.0 °C, the PCR amplicons are incubated at 70.0 °C for cross-hybridization. The PCR temperature is raised to the T_c_ (81.5 °C) to preferentially denature the heteroduplex ds DNA wt-mt compared to ds DNA wt-wt or mt-mt, followed by the annealing temperature (60.0 °C), and then increased to 72.0 °C for primer extension, thus preferentially amplifying the mutation-containing alleles.

Depending on the sequence context and position of the mismatch, T_m_ can change 0.2–1.5 °C for a sequence of ≤200 nt. T_c_ is strongly dependent on DNA sequence, and it is determined practically for each specific sequence. To determine T_c_ for both COLD-PCR of P1 and P2 fragments, wild-type PCR amplicons were first amplified by SYBR Green real time PCR using TOPreal™qPCR 2X PreMIX (Enzynomics, Incheon, Korea) containing respective primers at the same PCR condition as described above for conventional PCR and then were subjected to melting-curve analysis (ramping at 0.2 °C/s from 65 °C to 97 °C, continuous with 5 readings/°C) to identify the T_m_ values on Light Cycler 96 (Roche Diagnostics, Mannheim, Germany). Determination of T_c_ values was obtained by using the wt control as a template using the following real time PCR condition: 95 °C for 10 min; 35 cycles of T_x_ for 15 s, 60 °C for 20 s, and 72 °C for 20 s, where T_x_ values are chosen based on T_m_ values of P1 and P2 fragments, starting from T_m_ value + up to 0.7 °C to lower temperatures until no more real time PCR products are obtained. T_c_ = lowest T_x_ value which still allows amplification, 0.4 °C. After the T_c_ values were determined, the condition for COLD-PCR was optimized as follows: 95 °C for 10 min; 10 preamplification cycles: 95 °C for 15 s, 60 °C for 20 s, and 72 °C for 20 s; and 35 cycles of 95 °C for 15 s, 70 °C for 90 s (hybridization), T_c_ for 15 s, 60 °C for 20 s, and 72 °C for 20 s. The schematic workflow for the COLD-PCR assay is presented in Figure 1. The mt and wt controls were mixed at a molar ratio of 1%, 5%, and 10% mt/wt and adjusted to a total of 1.10^6^ copies/mL for use as mt:wt standards because this concentration is close to the minimal viral load (6.10^5^ IU/mL, equivalent to 3.10^6^ copies/mL) among all samples collected in this study. The mixed DNA was then subjected to conventional PCR-Sanger sequencing and COLD-PCR-Sanger sequencing to compare the sensitivities of the two methods. After that, the optimized COLD-PCR condition was applied for P1 and P2 fragment amplifications using the extracted DNA from 142 collected patient blood samples and 2X TOPsimple™ PreMIX-HOT (Enzynomics, Incheon, Korea) containing the respective primers. The obtained amplified 193 bp P1 fragment and 197 bp P2 fragment were also checked by agarose-gel electrophoresis before sending for Sanger DNA sequencing.

### 2.6. Other Molecular and Serological Assays

HBV viral load was measured by COBAS Ampli-Prep/COBAS Taqman HBV Test (Roche Diagnostics, Indianapolis, IN, USA). HBeAg level was measured by electrochemiluminescence immunoassay “ECLIA” using commercial kits VIDAS HBE/HBET (BioMerieux, Marcy-I’Etoile, France). Aspartate Transaminase (AST) and Alanine aminotransferase (ALT) were measured using an ultraviolet absorption spectrophotometry assay (Beckman Coulter, Brea, CA, USA).

### 2.7. DNA Sequence and Statistical Analysis

The DNA sequences of PCR or COLD-PCR products were aligned and translated to the consensus amino acid sequence to identify the NA-resistance substitutions by SnapGene software. The low rate mt signals were confirmed by Poly Peak Paster software. NA-drug resistant mt was classified into classical and nonclassical based on the full or partial validation of their drug-resistance-inducing mechanism, respectively. All sequences with completely replaced mt were further determined based on an HBV-drug resistance interpretation online tool of Max Planck Institute for Informatics for classical mt, e.g., rtL180M, rtA181V/T, rtT184G/L, rtA194T, rtS202I/G, rtM204I/V, rtN236T, and rtM250I/V, and based on updated references for nonclassical/putative mt, e.g., V207I/M/L, rtS213T, rtL229V, rtI233V, rtP237T, rtN238A/K/T, and rtS256G [15,16,24,25,26,27,28,29,30,31,32,33,34]. This classification method has been reviewed by Zang et al. [35] and Zoulim et al. [27] and was cited as the basis for classification in the HBV database 2020. The HBV genotype was analyzed using the same above online tool. All statistical analysis was performed using the SPSS software, version 20. Pearson’s chi-square or Fisher’s extract tests were used to determine whether there were any differences in frequency between groups. *p* < 0.05 was considered statistically significant. The odds ratio (OR) and 95% confidence intervals (CI) of OR were used to determine associations between the presence of mutations and any clinical features.

### 2.8. Real-Time PCR for Confirming NA Drug-Resistance-Associated mt

The two DNA clinical samples carrying mt rtV207I (G750A) and mt rtV207I (G750T) at low levels of mt/wt were used for detection of the mt by real-time PCR using the Forward-HBV-P1/Reverse-HBV-P1 primers and Lock Nucleic Acid (LNA) fluorescent probes (IDT, IA, USA). The LNA probes were labelled at the 5′-end with HEX (6-carboxy-2′,4,4′,5′,7,7′–hexachlorofluorescein) reporter and at the 3′ end with IBFQ (Iowa black fluorescence quencher), matching the mt (A/T) while forming a G:A/T mismatch with the wt sequence. The probe sequences were as follows: (G750A probe) 5′-HEX/ATGG+ATGATA+T+A***+***GT+ATTGG/3′-IABkFQ (nt 739–757); (G750T probe) 5′-HEX/ATGG+ATGATA+T+T***+***GT+ATTGG/3′-IABkFQ (nt 739–757), where “+” indicates the position of LNA and the underline indicates a mismatched nucleotide. Real-time PCR was performed on Light Cycler 96 (Roche Diagnostics, Mannheim, Germany) for the samples in parallel with the positive control (5% mt/wt adjusted at total 1.10^6^ copies/mL) and the negative control (wt at 1.10^6^ copies/mL). The 25 µL reaction mixtures contained 12.5 µL of TOPreal™ qPCR 2XPreMIX (Enzynomics, Incheon, Korea), 400 nM of each primers, 40 nM of probes, and 5 µL of the DNA template. Real-time PCR was run at conditions of 95 °C for 10 min, 45 cycles of 95 °C for 15 s and 60 °C for 60 s accompanied by its software to analyze the relative fluorescent intensity (≥0.3) and threshold cycles (C_t_ ≤ 40) for positive samples.

## 3. Results

### 3.1. Identification of Critical Denaturation Temperatures 

**Figure 2:**
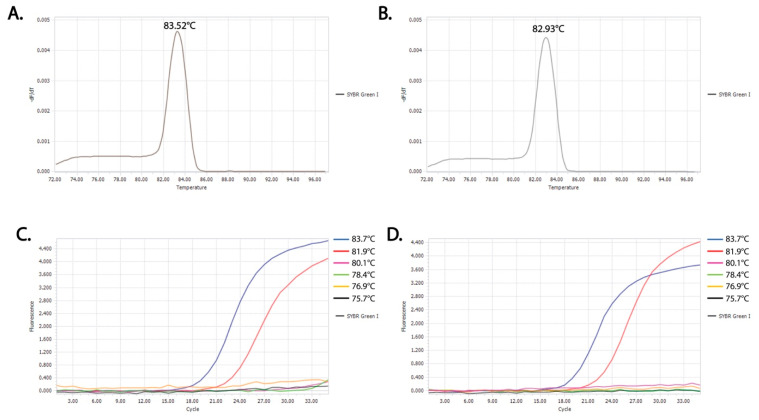
SYBR Green Real time PCR result for the determination of T_c_: Melting curve analysis of PCR products for the detection of T_m_ for P1 fragment (**A**) and P2 fragment (**B**) and fluorescent amplification curves at different T_x_ values (83.7 °C, 81.9 °C, 80.1 °C, 78.4 °C, 76.9 °C, and 75.7 °C) to identify T_c_ values for COLD-PCR assays of P1 fragment (**C**) and P2 fragments (**D**).

The T_m_ values for the P1 and P2 fragments containing major NA-drug resistance mt in HBV RT, as determined from the melting curve, were 83.52 °C and 82.93 °C, respectively (Figure 2A,B). The T_x_ values for this experiment were set automatically by the real-time PCR system with a serial six temperature points including 83.7 °C, 81.9 °C, 80.1 °C, 78.4 °C, 76.9 °C, and 75.7 °C. Fluorescent curves shown in Figure 2C,D indicated that, for both P1 and P2 fragments, 81.9 °C was the lowest temperature allowing homoduplex DNA templates to be denatured and PCR products to be amplified and that 80.1 °C was the highest temperature completely inhibiting the denaturing step of the homoduplex DNA templates. The T_c_ values should be within the range from 80.1 °C to 80.9 °C (80.1 °C < T_c_ < 81.9 °C). After optimization, the value of 81.5 °C (81.9 °C − 0.4 °C) was picked as the T_c_ for COLD-PCR assay of both P1 and P2 fragments (homoduplex ds DNA wt-wt and mt-mt hardly unwinds; heteroduplex ds DNA wt-mt can successfully unwind) (Figure 1). 

### 3.2. Sensitive DNA Sequencing Coupled with COLD-PCR Amplification for Detection of NA-Resistance mt

**Figure 3:**
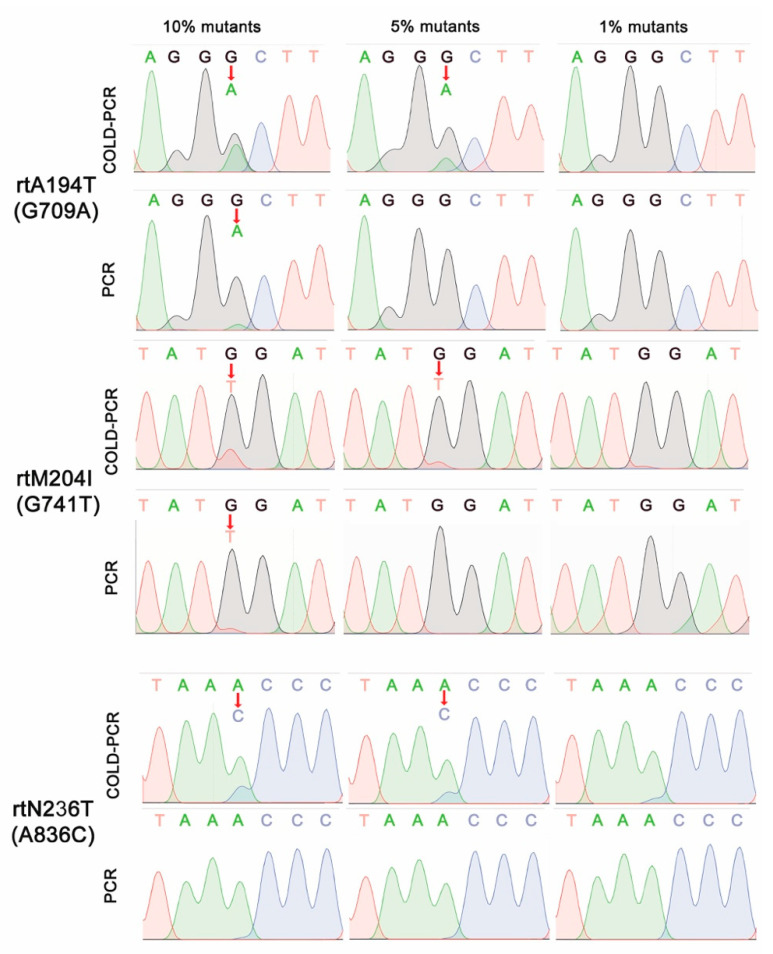
Conventional PCR and COLD-PCR evaluations for 1%, 5%, and 10% mt abundance following dilution of plasmids carrying rtA194T (G709T) mt in domain B, rtM204I (G741T) mt in domain C, and rtN236T (A836C) mt in domain D of HBV RT into the wt plasmid: the red arrows are positions of mutated bases in the DNA sequencing chromatograms.

We then compared the sensitivity of the COLD-PCR method (with denaturation at 81.5 °C) for amplifying the P1 and P2 fragments, containing major NA–resistance single-point mt, and the conventional PCR method (with a denaturation at 95 °C) for amplifying the B, C, D, and E domains using a mixture of controls at molar proportions of 1%, 5%, and 10% mt/wt. Figure 3 shows nucleotide peaks of conventional PCR and COLD-PCR products using templates as the mt:wt mixture controls of the wt and individual mt including rtA194T, rtM204I, and rtN236T locating at the respective domains B, C, and D of HBV RT. It appeared that conventional PCR allows detection of drug-resistance mt at only 10% mt/wt and that the mt peak created by this method was always lower than that by the COLD-PCR method. In contrast, the COLD-PCR method successfully enriched the mt population, resulting in higher mt peaks and a 5% mt/wt ratio detection level for the rtA194T (G709A), rtM204I (G741T), and rtN236T (A836C) mt. None of the methods could detect 1% mt in a mixed mt:wt population. These results revealed that the optimized COLD-PCR method was more sensitive than conventional PCR for detecting minor mt fractions. We, therefore, used the COLD-PCR method for screening mt in clinical specimens and the conventional PCR method only for comparison.

### 3.3. Characteristics of Subjects

The characteristics of the 142 treatment-naive CHB patients with median age 62.8 months enrolled in this study are presented Table 1; 43.7% of them were females. ALT and AST serum levels were 160.1 ± 21.2 IU/L and 153.4 ± 25.3 IU/L (median ± standard error), and HBV DNA serum levels had a range of 6.10^5^–1.10^9^ IU/mL; 88.7% were HBeAg positive, 76.8% were HBV genotype B, and 23.2% were HBV genotype C.

### 3.4. Rate and Association of NA-Resistance Mutations with Characteristics and Subclinical Index of Treatment-Naive CHB Children

Using the optimized COLD-PCR combined Sanger DNA sequencing method, both classical and nonclassical mt associated with NA drug resistance were found in 94 (66.2%) of the 142 clinical specimens. As shown in the Table 1, we could not find any association of possible NA-resistance mt carrier with patients characteristics and subclinical indexes, such as age (≤60 vs. >60 months; OR_mt_: 0.812; 95% CI: 0.403–1.633; *p*: 0.558), gender (male vs. female; OR_mt_: 1.005; 95% CI: 0.499–2.027; *p*: 0.988); ALT and AST levels (≤40 vs. >40 IU/L; OR_mt_: 1.722 and 1.273; 95% CI: 0.817–3.629 and 0.578–2.801; *p*: 0.151 and 0.549), HBeAg presence (positive vs. negative; OR_mt_: 0.621; 95% CI: 0.189–2.041; *p*: 0.429), or viral load (<10^8^ IU/mL vs. >10^8^ IU/mL; OR_mt_: 0.968; 95% CI: 0.422–2.219; *p*: 0.938). Among the 94 cases, there were 66 cases (46.5%) having mt associated with LMV resistance, 24 cases (16.9%) having mt associated with ADF resistance, and 4 cases (2.8%) having mt associated with more than one drug resistance or reduced susceptibility (LMV + ADF × 2 cases; LMV + ETV × 1 case; LMV + ADF + ETV × 1 case) (Table 2 and Table 3). Further analysis of the mt associated with only LMV or ADF resistance showed no correlation between LMV- or ADF-associated mt and age, gender, ALT, AST, HBeAg, and viral load. However, it was interesting to see that there were up to 65/66 genotype B cases (98.5%) resistant to LMV and up to 23/24 genotype C cases (95.8%) resistant to ADF. These data indicate that there is a significantly higher frequency of LMV- and ADF-associated mt in genotypes B and C, respectively (OR_LMV/ADF_: 1495.000; 95% CI: 89.800–24889.032; *p* < 0.001).

### 3.5. Profile of Mutations in Treatment-Naive CHB Children

Regarding NA drug-resistance-associated mt, 94 patients with 9 sites were found to be mutated, including 2 classical mt sites (rtL180 and rtM204) and 7 nonclassical mt sites (rtV207, rtS213, rtL229, rtI233, rtP237, rtN238, and rtS256) (Table 3). More specifically, 85 cases (59.9%) had mt causing a single amino acid substitution associated with LMV and/or ADF resistance, the most frequent being rtV207M (54 cases; 38.0%), followed by rtN238T (14 cases; 9.9%); rtN238A (8 cases; 5.6%); rtV207L (2 cases; 1.4%); rtS213T (2 cases; 1.4%); rtS256G (2 cases; 1.4%); and rtL229V, rtP237T, and rtN238K in 1 case each (0.7%). Multiple-point mt associated with LMV or ADF resistance were observed in 9 cases (6.3%), 6 cases carried double mt (rtV207M + rtS213T; rtV207M + rtL229V; rtV207M + rtI233V; rtV207I + rtV207M × 2 cases; rtN238A + rtS256G), 2 cases carried triple mt (rtL180M + rtM204I + rtN238T; rtV207M + rtS213T + rtS256G), and 1 case carried quadruple mt (rtL180M + rtM204V + rtV207I + rtV207M). Among the two later cases, there were 3 classical mt including rtM204V/I (primary drug resistance mt) and rtL180M (compensatory/secondary mt). These cases confer reduced sensitivity to ETV besides LMV-ADF resistance (Table 3).

Among these above 94 cases, there were 11 cases carrying additional mt that are not related to drug resistance, such as rtI187V (+ rtV207M) × 2 cases, rtI187V (+ rtS213T), rtI187V (+ rtP237T), rtI187V (+ rtL229V), rtI187V (+ rtV207M + rtS213T + rtS256G), rtK212T (+ rtV207I + rtV207M), rtV253I (+ rtV207M), rtV253I (+ rtN238A) × 2 cases, and rtL235V (+ rtV207M + rtL229V). In addition, 23 cases had only mt that are not related to drug resistance, such as rtI187V × 20 cases, rtV253I, rtS256C, and rtI187V + rtN248H.

### 3.6. Verification of Representative Samples Having Minor mt Peaks

**Figure 4:**
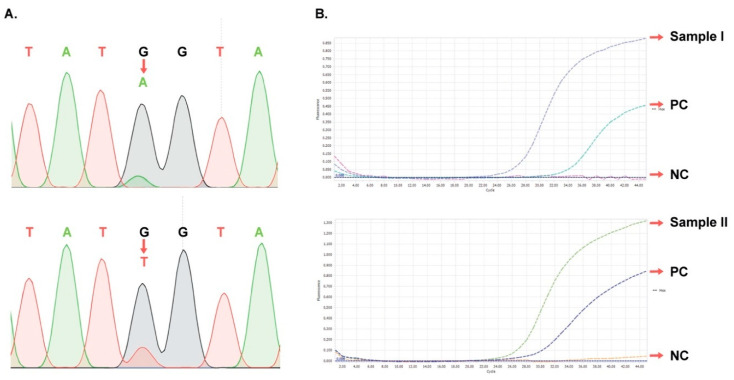
Detection of mt rtV207I (G750A) and mt rtV207I (G750T) in two clinical samples by COLD-PCR combined DNA sequencing and then confirmation by real-time PCR using an Lock Nucleic Acid (LNA) probe: (**A**) DNA sequencing chromatograms for samples I and II carrying the mt rtV207I (G750A) (upper panel) and mt rtV207I (G750T) (lower panel), respectively. The red arrows are the positions of mutated bases. (**B**) Fluorescent real-time PCR amplification curves of samples I and II (upper panel) and of sample II (lower panel), respectively. PC, positive control (5% mt/wt); NC, negative control (wt only).

During analysis of the sequencing chromatograms for 92 cases having NA drug-resistance-associated mt, we found 2 representative cases carrying mt rtV207I (G750A) and mt rtV207I (G750T) which have the minor (mt) peaks constituting ≤ 20% of major (wt) peaks at rt positions. As shown in Figure 4A, at the nt 750, the mt peaks (A/T) appeared as tiny peaks compared to the wt peak (G), indicating that the levels of mt/wt are close to detection limit (5% mt/wt). Thus, we decided to verify these two mt by real-time PCR using the two LNA probes were designed specifically at the mt site. As shown in Figure 4B, the intensities of fluorescent curves in both samples were much higher than that of the control (0.88 vs. 0.46 for G750A; 1.32 vs. 0.85 for G750T) and that the C_t_ values of both samples were smaller than that of the control (26.14 vs. 33.96 for mt G750A; 25.51 vs. 28.47 for G750T). Our data confirms that the two samples are really positive mt rtV207I (G750A) and mt rtV207I (G750T). There were non-detectable HEX signals in the negative control, confirming that there was no background of nonspecific HEX signals due to high mt-specificity of the LNA probes.

## 4. Discussion

Determination of mt in the RT region of HBV has been widely conducted to monitor NA-drug resistance in long-term treatment for patients with CHB, contributing to important insights in drug selection for clinicians. However, the application of early detection of NA resistance-associated mt for treatment-naive CHB pediatric patients is still limited. In this study, a total of 142 treatment-naive CHB children in northern Vietnam were selected for detection of drug resistance-associated mt using the sensitive COLD-PCR combined with Sanger DNA sequencing at a 5% mt/wt ratio sensitivity. Although mt quasi-species less than 5% were non-detectable using the technique, this method was chosen over next-generation sequencing owing the limited funding for this study as well as to its potentials in terms of high sensitivity, cost-effectiveness, and short run period in clinical diagnostic application in low- and middle-income countries, including Vietnam. With the design of COLD-PCR for the amplification of P1 and P2 fragments, corresponding to the B, C, D, and E domains in HBV RT, the developed method can be applied for sensitive detection of major NA-drug resistance mutations, including three mt (rtV173L, rtL180M, and rtM204I/V) that are associated not only with LMV and ETV resistance but also with vaccine escape [12,14]. Additional mt including rtS256G for resistance to LMV and rtL229V, rtI233V, rtP237T, and rtN238A/K/T for resistance to ADF were detected based on the work of Liu and his colleagues [21]. To confirm the reliability of mt detection in clinical samples by the COLD-PCR method, we cross-checked the two representative samples having the lowest mt peaks constituting ≤20% of the wt peak heights based on real-time PCR assay using Taqman LNA probes. Our data confirms that these two samples are actually positive with mt rtV207I (G750A) and mt rtV207I (G750T). Although we were not able to design LNA probes for all mt found in this study, the data suggest that the other remaining mt having mt peaks constituting >20% of the wt peak heights detected by COLD-PCR combined Sanger DNA sequencing are reliable. 

Our results showed that the rate of mt associated with NA drug resistance is rather high, up to 66.2%, with nonclassical mt contributing the most (64.8%). Fortunately, most mt were LMV and ADF resistance only and only two cases were ETV intermediate-level resistance, indicating that ETV and TDF are still effective for the treatment of such patients. The data also suggest that NA-resistance mt analysis is necessary before treatment of children using LMV or ADF. The high rate of mt in these children may be due to an antiviral drug resistance mother-to-child transmission or to the high LMV and ADF resistance of HBV genotypes that are circulating in Vietnam. This rate is much higher than that reported on treatment-naive adult patients with CHB in recent years. In the review by Choi et al. [16], there have been respective rates of 26.8% (26/96), 16.8% (60/357), 8.9% (24/269), 7.3% (7/96), 5% (13/286), and 1% (2/198) found in Indian, Chinese, Indonesian, Italian, and Brazilian treatment-naive patients carrying drug resistance-associated mt. Such a difference is due to a number of factors, which include (i) differences in detection methods and patient characteristics (previous study has applied conventional PCR for DNA sequencing, INNO-LiPA line assay); (ii) analysis methods for classification of NA drug mt (some reports analyze only classical mt), (iii) NA-resistant levels of HBV genotypes circulating in each country, and (iv) characteristics of patients participating in the study. 

Regarding such a high frequency of nonclassical mt found in treatment-naïve CHB children, we believe that our results are rational. The classical mt are mostly found in patients that have gone under treatment, in which the drug treatment acts as the favorable selection pressure for drug-resisting (classical) mt. These primary classical mt, on the other hand, have lowered reverse-transcription efficiency until the compensation of a secondary mt that alleviates the reduced reverse-transcription rate [19,36,37]. Therefore, under conditions without drug-treatment, as in our subjects, the primary classical mt have no evolutionary advantages and are selected against. This results in only 2 cases of detectable quasi-species of classical mt in our data. Our findings on the high frequency of nonclassical mt are consistent with previous reports from other groups [17,38]. For example, in a study by Nguyen et al. (2009) on 472 samples collected from treatment-naïve American adults using conventional PCR combined Sanger DNA sequencing, 389 cases (82.4%) were found to not carry any NA-drug resistance mt, 79 cases (16.7%) were found with rtV207M, and only 4 cases (0.9%) were found with other “putative” mt [38]. While our reported spectrum of mt in treatment-naïve resembles that of Nguyen et al., with the majority of mt being rtV207M, we also speculated that, with our improved COLD-PCR assay, several mt that were presumably assigned as non-mutation in the Nguyen et al. study were detected accordingly. This resulted in additional nonclassical mt in our study.

Distribution of HBV drug resistance-associated mt was found mainly in genotypes B and C, which is in good agreement with a recent review by Choi and his colleagues [16] regarding several studies that included treatment-naïve Chinese and Japanese patients infected with genotypes B and C. Our data indicates that 65/66 genotype B cases carried LMV-associated mt with the most frequent rtV207, while 23/24 genotype C cases carried ADF-associated mt with the most frequent rtN238. This data is consistent with a previous study by Zang et al. [35] about the high frequency of LMV-associated mt found in HBV genotype B of CHB Chinese patients. Moreover, our study is also consistent with another study by Xu et al. (2015) on 168 treatment-naïve Chinese adults that the majority of mutants were nonclassical ones found in genotypes B and C, including rtV207M [17]. However, our data differs from a recent report by Mokaya et al. [12] concerning the random distribution of LMV and ADF resistance-associated mt in genotypes A, D, and E found in African CHB patients. This shows regional dependence properties and a predisposition to NA drug-resistance mt in HBV, probably due to the HBV polymerase non-proofreading nature and high rate of replication. To account for the dominance of nonclassical mt in treatment-naïve patients, we speculated that, under conditions without drug-treatment and while the classical primary mutants have a lowered replication efficiency, the nonclassical secondary mutants have slight advantages over both the wild-type and drug-resistance-inducing mutants, thus resulting in a high frequency of these mutants in all studies on treatment-naïve patients. This was addressed in a report by Zollner et al. (2005) on the nonclassical rtV207M/I mt—the most frequent nonclassical mt found in treatment-naïve patients—that rtV207M/I is regarded as a compensatory or secondary mt which led to suppression of the wt and predominance of the mt, possibly due to an increased replication competence [27].

Regarding the profile of mt associated with NA drug resistance, the majority was single amino acid substitution. In our data, the LMV-associated rtV207M was the most abundant (38.0%), followed by ADF-associated rtN238T (9.9%), ADF-associated rtN238A (5.6%), LMV-associated rtV207L (1.4%), LMV-associated rtS213T (1.4%), LMV-associated rtS256G (1.4%), LMV-associated rtL229V (0.7%), ADF-associated rtN238K (0.7%), and ADF-associated rtP237T (0.7%). We also detected rtI187V with a high prevalence of 14.1%. This mt is not associated with NA resistance but has been reported to reduce the rate of viral replication [39]. Multiple-point mt are found to be rare, including rtV207M + rtV207I; rtV207M + rtS213T; rtV207M + rtL229V; rtV207M + rtI233V; rtN238A + rtS256G; rtL180M + rtM204I + rtN238T; rtV207M + rtS213T + rtS256G; and rtL180M + rtM204V + rtV207I + rtV207M. Among those mt, we found only 2 cases of respective HBV genotypes B and C that have rtM204V and rtM204I primary mt coexisting with rtL180M compensatory/secondary mt. Cross-resistance among some NA chemical groups have been reported by Yamada et al. [40], and our study also indicates that the 3 cases having 3 or 4 mt can be considered LMV resistant and ETV intermediate-level resistant. In such patients, previous studies have reported that the effectiveness in treatment by ADF and ETV are significantly reduced [37,41]. Thus, with these three cases, TDF therapy can be suggested as an alternative treatment. 

Additionally, 6 mt including rtI187V, rtK212T, rtL235V, rtN248H, rtV253I, and rtS256C were found either independently (23 cases) or combined with NA drug-resistance-associated mt (11 cases), but their role in drug resistance in previous reports has not been determined.

In conclusion, our study for the first time reports the high prevalence of antiviral-drug resistance-associated mt in treatment-naive Vietnamese children with CHB using a sensitive COLD-PCR combined with DNA Sanger method. The mt occurred mainly in genotypes B and C, which are resistant to LMV and ADF, respectively. The majority of them are single-point mt, and only a few are multiple-point mt, which may determine LMV resistance and ETV-reduced susceptibility. These data suggest that TDF is still an effective treatment for all cases and that NA-resistance mt analysis is useful before initiation of antiviral therapy for the treatment-naive CHB pediatric patients, especially when LMV and ADF are intended for prescription. Doctors may consider prescribing LMV instead of ETV and ADF instead of TDF for the treatment of CHB in children between 2–12 years of age and children above 12 years of age, respectively. This can be done if patients do not carry LMV or ADF resistance-associated mt and especially when ETV and TDF stock or the budget for treatment is limited. This also helps to reduce the risk of future ETV and TDF resistance. In addition, the developed method is potentially applicable to the detection of mutations in other regions, such as vaccine escape mt in the gene coding for HBV protein S.

## Figures and Tables

**Table 1:** Clinical features of treatment-naïve children with chronic hepatitis B.

Characteristic	Total *n (%)*	Min-Max	Average	NA-Drug Resistance-Associated mt *n*	Other mt and wt *n*	Odds Ratio (95% CI)	*p* Value
**Age (months)**		2–183	62.8 ± 3.8				
≤60	75 (52.8)			48	27	0.812 (0.403–1.633)	*0.558*
>60	67 (47.2)			46	21		
**Gender**							
Male	80 (56.3)			53	27	1.005 (0.499–2.027)	*0.988*
Female	62 (43.7)			41	21		
**AST (IU/L)**		15.5–2161.7	153.4 ± 25.3				
≤40	40 (28.2)			28	12	1.273 (0.578–2.801)	*0.549*
>40	102 (71.8)			66	36		
**ALT (IU/L)**		11.0–1849.7	160.1 ± 21.2				
≤40	53 (37.3)			39	14	1.722 (0.817–3.629)	*0.151*
>40	89 (62.7)			55	34		
**HBeAg (+/−)**							
Positive	126 (88.7)			82	44	0.621 (0.189–2.041)	*0.429*
Negative	16 (11.3)			12	4		
**HBV DNA level (IU/mL)**		6.10^5^–1.10^9^	6.10^8^				
<10^8^	32 (22.5)			21	11	0.968 (0.422–2.219)	*0.938*
≥10^8^	110 (77.5)			73	37		
**Genotype HBV**							
B	109 (76.8)			68	41	0.447 (0.178–1.121)	*0.081*
C	33 (23.2)			26	7		

HBeAg, hepatitis B e antigen; HBV, hepatitis B virus; AST, aspartate aminotransferase; ALT, alanine aminotransferase; CI, confidence interval; IU, international unit.

**Table 2:** Association of Lamivudine (LMV) and Adefovir dipivoxil (ADF) resistance mutations with patients characteristics.

Characteristics	Total *n*	LMV Resistance-Associated mt *n*	ADF Resistance-Associated mt *n*	Odds Ratio (95% CI)	*p* Value
**Age (months)**					
≤60	47	36	11	1.418 (0.555–3.623)	*0.464*
>60	43	30	13		
**Gender**					
Male	51	37	14	0.911 (0.354–2.347)	*0.847*
Female	39	29	10		
**AST (IU/L)**					
≤40	26	20	06	1.304 (0.451–3.775)	*0.624*
>40	64	46	18		
**ALT (IU/L)**					
≤40	36	26	10	0.910 (0.352–2.353)	*0.846*
>40	54	40	14		
**HBeAg (+/−)**					
Positive	78	57	21	0.905 (0.223–3.666)	*1.000*
Negative	12	09	03		
**HBV DNA level (IU/mL)**					
<10^8^	19	14	05	1.023 (0.324–3.226)	*0.969*
≥10^8^	71	52	19		
**HBV genotype**					
B	66	65	1	1495.000 (89.800–24889.032)	*< 0.001*
C	24	1	23		

**Table 3:** Profile of Nucleos(t)ide-analogue (NA) drug resistance-associated mutations in treatment-naïve children with chronic hepatitis B.

Patients		Mutation Types	Resistance/Susceptibility to NA Drug				References
*n*	%		LMV	ADF	TDF	ETV	
**Single-Point Mutation**							
54	38.0	rtV207M	R	S	S	S	[25]
2	1.4	rtV207L	R	S	S	S	[32]
2	1.4	rtS213T	R	S	S	S	[25]
1	0.7	rtL229V	R	S	S	S	[31]
1	0.7	rtP237T	S	R	S	S	[28,33]
8	5.6	rtN238A	S	R	S	S	[25]
1	0.7	rtN238K	S	R	S	S	[24]
14	9.9	rtN238T	S	R	S	S	[28,30]
2	1.4	rtS256G	R	S	S	S	[15,16,26]
**Multi-Point Mutation**							
2	1.4	rtV207M + rtV207I	R	S	S	S	[25,27]
1	0.7	rtV207M + rtS213T	R	S	S	S	[25]
1	0.7	rtV207M + rtL229V	R	S	S	S	[25,31]
1	0.7	rtV207M + rtI233V	R	R	S	S	[25,29,34]
1	0.7	rtN238A + rtS256G	R	R	S	S	[15,16,25,26]
1	0.7	rtL180M + rtM204I + rtN238T	R	R	S	I	[19,23,28]
1	0.7	rtV207M + rtS213T + rtS256G	R	S	S	S	[15,16,25,26]
1	0.7	rtL180M + rtM204V + rtV207I + rtV207M	R	S	S	I	[19,23,25,27]
Total							
94 (66.2%)			70 (49.3%)	27 (19.0%)	0 (0%)	0 (0%)	

ADF, adefovir dipivoxil; CHB, chronic hepatitis B; ETV, entecavir; HBeAg, hepatitis B e antigen; HBV, hepatitis B virus; I, intermediate/reduced susceptibility; LMV, lamivudine; R, resistance; S, susceptible; TDF, tenofovir disoproxil fumarate.

## References

[1] Xu D.-Z., Yan Y.-P., Choi B.C.K., Xu J.-Q., Men K., Zhang J.-X., Liu Z.-H., Wang F.-S. (2002). Risk factors and mechanism of transplacental transmission of hepatitis B virus: A case-control study. J. Med. Virol..

[2] Wang Z., Zhang J., Yang H., Li X., Wen S., Guo Y., Sun J., Hou J. (2003). Quantitative analysis of HBV DNA level and HBeAg titer in hepatitis B surface antigen positive mothers and their babies: HBeAg passage through the placenta and the rate of decay in babies. J. Med. Virol..

[3] Wiseman E., Fraser M.A., Holden S., Glass A., Kidson B.L., Heron L.G., Maley M.W., Ayres A., Locarnini S.A., Levy M.T. (2009). Perinatal transmission of hepatitis B virus: An Australian experience. Med. J. Aust..

[4] Dusheiko G. (2012). Interruption of mother-to-infant transmission of hepatitis B: Time to include selective antiviral prophylaxis?. Lancet.

[5] Lok A.S.F., McMahon B.J. (2009). Chronic hepatitis B: Update 2009. Hepatology.

[6] Sokal E.M., Paganelli M., Wirth S., Socha P., Vajro P., Lacaille F., Kelly D., Mieli-Vergani G. (2013). Management of chronic hepatitis B in childhood: ESPGHAN clinical practice guidelines. J. Hepatol..

[7] European Association for the Study of the Liver (2017). EASL 2017 Clinical Practice Guidelines on the management of hepatitis B virus infection. J. Hepatol..

[8] Komatsu H., Inui A., Fujisawa T. (2017). Pediatric hepatitis B treatment. J. Thorac. Dis..

[9] Terrault N.A., Lok A.S.F., McMahon B.J., Chang K., Hwang J.P., Jonas M.M., Brown R.S., Bzowej N.H., Wong J.B. (2018). Update on prevention, diagnosis, and treatment of chronic hepatitis B: AASLD 2018 hepatitis B guidance. Hepatology.

[10] Ohno M., Otsuka M., Kishikawa T., Yoshikawa T., Takata A., Koike K. (2015). Novel therapeutic approaches for hepatitis B virus covalently closed circular DNA. World J. Gastroenterol..

[11] Fares M.A., Holmes E.C. (2002). A Revised Evolutionary History of Hepatitis B Virus (HBV). J. Mol. Evol..

[12] Mokaya J., McNaughton A.L., Hadley M.J., Beloukas A., Geretti A.-M., Goedhals D., Matthews P.C. (2018). A systematic review of hepatitis B virus (HBV) drug and vaccine escape mutations in Africa: A call for urgent action. PLOS Negl. Trop. Dis..

[13] Tacke F., Kroy D.C. (2016). Treatment for hepatitis B in patients with drug resistance. Ann. Transl. Med..

[14] Park E.-S., Lee A.R., Kim D.H., Lee J.-H., Yoo J.-J., Ahn S.H., Sim H., Park S., Kang H.S., Won J. (2019). Identification of a quadruple mutation that confers tenofovir resistance in chronic hepatitis B patients. J. Hepatol..

[15] Yamani L.N., Yano Y., Utsumi T., Wasityastuti W., Rinonce H.T., Widasari D.I., Juniastuti, Lusida M.I., Soetjipto, Hayashi Y. (2017). Profile of Mutations in the Reverse Transcriptase and Overlapping Surface Genes of Hepatitis B Virus (HBV) in Treatment-Naïve Indonesian HBV Carriers. Jpn. J. Infect. Dis..

[16] Choi Y.-M., Lee S.-Y., Kim B.-J. (2018). Naturally occurring hepatitis B virus reverse transcriptase mutations related to potential antiviral drug resistance and liver disease progression. World J. Gastroenterol..

[17] Xu J., Wu B., Wang J.H., Huang L., Wang D.Y., Zhao L., Zhao G., Wang Y. (2015). Pre-existing mutations in reverse transcriptase of hepatitis B virus in treatment-naive chinese patients with chronic hepatitis B. PLoS ONE.

[18] Zhang X., Chen X., Wei M., Zhang C., Xu T., Liu L., Xu Z. (2019). Potential resistant mutations within HBV reverse transcriptase sequences in nucleos(t)ide analogues-experienced patients with hepatitis B virus infection. Sci. Rep..

[19] Lok A.S., Zoulim F., Locarnini S., Bartholomeusz A., Ghany M.G., Pawlotsky J.-M., Liaw Y.-F., Mizokami M., Kuiken C. (2007). Antiviral drug-resistant HBV: Standardization of nomenclature and assays and recommendations for management. Hepatology.

[20] Li J., Wang L., Mamon H., Kulke M.H., Berbeco R., Makrigiorgos G.M. (2008). Replacing PCR with COLD-PCR enriches variant DNA sequences and redefines the sensitivity of genetic testing. Nat. Med..

[21] Liu C., Lin J., Chen H., Shang H., Jiang L., Chen J., Ye Y., Yang B., Ou Q. (2014). Detection of Hepatitis B Virus Genotypic Resistance Mutations by Coamplification at Lower Denaturation Temperature-PCR Coupled with Sanger Sequencing. J. Clin. Microbiol..

[22] Wong D.K.-H., Tsoi O., Huang F.-Y., Seto W.-K., Fung J., Lai C.-L., Yuen M.-F. (2014). Application of Coamplification at Lower Denaturation Temperature-PCR Sequencing for Early Detection of Antiviral Drug Resistance Mutations of Hepatitis B Virus. J. Clin. Microbiol..

[23] Zoulim F., Locarnini S. (2009). Hepatitis B Virus Resistance to Nucleos(t)ide Analogues. Gastroenterology.

[24] Mahabadi M., Norouzi M., Alavian S.M., Samimirad K., Azad T.M., Saberfar E., Mahmoodi M., Ramezani F., Karimzadeh H., Malekzadeh R. (2013). Drug-Related Mutational Patterns in Hepatitis B Virus (HBV) Reverse Transcriptase Proteins From Iranian Treatment-Naïve Chronic HBV Patients. Hepat. Mon..

[25] He X., Wang F., Huang B., Chen P., Zhong L. (2015). Detection and analysis of resistance mutations of hepatitis B virus. Int. J. Clin. Exp. Med..

[26] Liu B.-M., Li T., Xu J., Li X.-G., Dong J.-P., Yan P., Yang J.-X., Yan L., Gao Z.-Y., Li W.-P. (2010). Characterization of potential antiviral resistance mutations in hepatitis B virus reverse transcriptase sequences in treatment-naïve Chinese patients. Antivir. Res..

[27] Zöllner B., Sterneck M., Wursthorn K., Petersen J., Schröter M., Laufs R., Feucht H.H. (2005). Prevalence, incidence, and clinical relevance of the reverse transcriptase V207I mutation outside the YMDD motif of the hepatitis B virus polymerase during lamivudine therapy. J. Clin. Microbiol..

[28] Bartholomeusz A., Locarnini S. (2006). Hepatitis B virus mutations associated with antiviral therapy. J. Med. Virol..

[29] Schildgen O., Sirma H., Funk A., Olotu C., Wend U.C., Hartmann H., Helm M., Rockstroh J.K., Willems W.R., Will H. (2006). Variant of Hepatitis B Virus with Primary Resistance to Adefovir. N. Engl. J. Med..

[30] Yim H.J., Hussain M., Liu Y., Wong S.N., Fung S.K., Lok A.S.F. (2006). Evolution of multi-drug resistant hepatitis B virus during sequential therapy. Hepatology.

[31] Locarnini S. (2008). Primary resistance, multidrug resistance, and cross-resistance pathways in HBV as a consequence of treatment failure. Hepatol. Int..

[32] Orlando R., Tosone G., Portella G., Veropalumbo E., D’Onofrio M., Piazza M. (2008). Prolonged Persistence of Lamivudine-resistant Mutant and Emergence of New Lamivudine-resistant Mutants Two Years After Lamivudine Withdrawal in HBsAg-positive Chronic Hepatitis Patient: A Case Report. Infection.

[33] Pollicino T., Isgrò G., Di Stefano R., Ferraro D., Maimone S., Brancatelli S., Squadrito G., Di Marco V., Craxì A., Raimondo G. (2009). Variability of reverse transcriptase and overlapping S gene in hepatitis B virus isolates from untreated and lamivudine-resistant chronic hepatitis B patients. Antivir.

[34] Schildgen O., Olotu C., Funk A., Zollner B., Helm M., Rockstroh J.K., Sirma H. (2010). Selection and Counterselection of the rtI233V Adefovir Resistance Mutation during Antiviral Therapy. J. Clin. Microbiol..

[35] Zhang Q., Liao Y., Chen J., Cai B., Su Z., Ying B., Lu X., Tao C., Wang L. (2016). Erratum: Corrigendum: Epidemiology study of HBV genotypes and antiviral drug resistance in multi-ethnic regions from Western China. Sci. Rep..

[36] Shaw T., Bartholomeusz A., Locarnini S. (2006). HBV drug resistance: Mechanisms, detection and interpretation. J. Hepatol..

[37] Tenney D.J., Rose R.E., Baldick C.J., Pokornowski K.A., Eggers B.J., Fang J., Wichroski M.J., Xu D., Yang J., Wilber R.B. (2009). Long-term monitoring shows hepatitis B virus resistance to entecavir in nucleoside-naïve patients is rare through 5 years of therapy. Hepatology.

[38] Nguyen M.H., Garcia R.T., Trinh H.N., Nguyen H.A., Nguyen K.K., Nguyen L.H., Levitt B. (2009). Prevalence of hepatitis B virus DNA polymerase mutations in treatment-naïve patients with chronic hepatitis B. Aliment. Pharmacol. Ther..

[39] Fan J., Wang Y., Xiong H., Guo X., Cheng Y.-C. (2014). Impact of the rtI187V polymerase substitution of hepatitis B virus on viral replication and antiviral drug susceptibility. J. Gen. Virol..

[40] Yamada N., Sugiyama R., Nitta S., Murayama A., Kobayashi M., Okuse C., Suzuki M., Yasuda K., Yotsuyanagi H., Moriya K. (2017). Resistance mutations of hepatitis B virus in entecavir-refractory patients. Hepatol. Commun..

[41] Lee Y.-S., Suh D.J., Lim Y., Jung S.W., Kim K.M., Lee H.C., Chung Y., Lee Y.S., Yoo W., Kim S. (2006). Increased risk of adefovir resistance in patients with lamivudine-resistant chronic hepatitis B after 48 weeks of adefovir dipivoxil monotherapy. Hepatology.

